# *Clostridium paraputrificum* Bacteremia Associated with Colonic Necrosis in a Patient with AIDS

**DOI:** 10.1155/2015/312919

**Published:** 2015-01-27

**Authors:** Takashi Shinha, Christiane Hadi

**Affiliations:** ^1^Department of Pathology, Microbiology and Immunology, Vanderbilt University Medical Center, 1301 Medical Center Drive, Nashville, TN 37232, USA; ^2^Department of Medicine, Indiana University School of Medicine, 3838 N Rural Hasbrook Building, Indianapolis, IN 46202, USA

## Abstract

*Clostridium* species are anaerobic Gram-positive rods that can cause a broad range of invasive infections in humans, including myonecrosis and bacteremia. Importantly, clostridial bacteremia is frequently associated with underlying medical conditions, such as colonic malignancy. Characterizing *Clostridium* spp. and understanding their associated clinical disease spectrum are paramount to provide optimal treatment, thereby decreasing morbidity and mortality especially in those with underlying debilitating comorbidities. *Clostridium paraputrificum* is an infrequently isolated *Clostridium* species and its clinical significance has not been well described. We herein report a case of bacteremia due to *C. paraputrificum* in a 65-year-old man with AIDS who developed acute colonic necrosis complicated by septic shock. We then review other cases of bacteremia associated with *C. paraputrificum* in the literature in addition to discussing the clinical significance of anaerobic bacteremia in general. To our knowledge, our report is the second case of *C. paraputrificum* bacteremia in a patient with AIDS.

## 1. Introduction

Anaerobic bacteria are a major component of the human microflora residing on mucous membranes. They can cause a wide array of infectious diseases following the breakdown of mucosal barriers at different anatomic sites. Among many* Clostridium* species,* Clostridium paraputrificum* is an unfamiliar infrequent isolate; therefore its clinical significance has not been well described. We report a case of bacteremia due to* C. paraputrificum* in a patient with AIDS to characterize the underlying disease spectrum as well as the clinical importance of* C. paraputrificum*-related bacteremia.

## 2. Case Report

A 65-year-old man with a history of acquired human immunodeficiency syndrome (AIDS) was in his usual status until three days prior to presentation, when he suddenly developed dizziness. He was seen in the emergency room at a public hospital in Indianapolis, where he was found to be persistently hypotensive. He was given intravenous fluid resuscitation and was hospitalized for further investigation. Human immunodeficiency virus (HIV) infection had been diagnosed 19 years before. The CD4 T-lymphocytes cell count at diagnosis was 525 cells per cubic millimeter, and the HIV viral load was 4500 copies per milliliter. The CD4 count one year to presentation was 248 per cubic millimeter, and the HIV viral load was 150,000 copies per milliliter. He had been noncompliant with his antiretroviral agents since the diagnosis of HIV infection. He also had a history of ischemic cardiomyopathy, atrial fibrillation, chronic orthostatic hypotension, diabetes mellitus, hepatitis B, hepatitis C, and end-stage renal disease for which he was receiving hemodialysis. The patient was taking didanosine, stavudine, and nevirapine for HIV intermittently. He was not on antiretroviral therapy on admission. Two days after admission, he developed severe abdominal pain followed by massive hematochezia. He subsequently developed hemodynamic instability which required the initiation of vasopressin and norepinephrine. An abdominal radiograph showed small bowel ileus and the colonic cutoff sign at the splenic flexure. A computer tomography scan of the abdomen showed diffuse colonic wall thickening. An emergent exploratory laparotomy revealed extensive colitis with necrosis which necessitated subtotal colectomy. On physical examination after surgery, the patient appeared critically ill. The temperature was 99.5°F (37.5°C), blood pressure 102/59 mm Hg, pulse 100 beats per minute, respirations 25 breaths per minute, and oxygen saturation 99% on FiO_2_ of 40%. His heart sounds were regular without murmurs and his lungs were clear to auscultation. There was a large abdominal midline incision. The abdomen was remarkably distended. There were three drainage tubes with serosanguinous fluid inside. A right sided colostomy was also in place. He had an arteriovenous fistula on the left arm. The remainder of physical examination was unremarkable.

**Figure 1:**
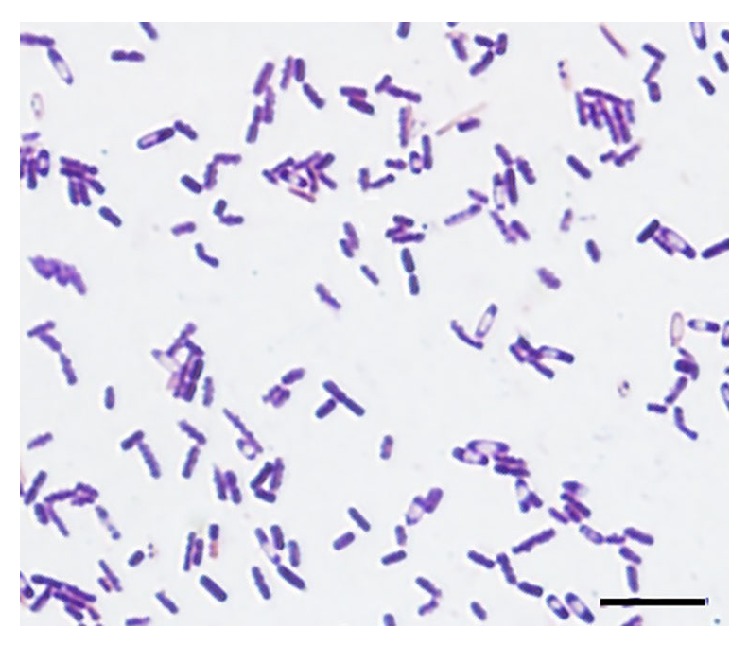
Gram stain of the blood culture showing Gram-positive rods with spores.

Laboratory studies revealed a white blood cell count of 13,100 cells/mm³ with 12% band forms, hemoglobin of 8.0 g/dL, and platelets of 96,000 per cubic millimeter. Other abnormal laboratory values included potassium 4.7 mmol/L, bicarbonate 18 mEq/L, urea nitrogen 33 mg/dL, creatinine 4.5 mg/dL, aspartate aminotransferase 100 U/L, total bilirubin 5.5 mg/dL, and lactic acid 10.0 mmol/L. The CD4 count was 10 per cubic millimeter, and the HIV viral load was 630,000 copies per milliliter. The patient was started on intravenous vancomycin and piperacillin/tazobactam. Caspofungin was added after colectomy. The patient remained in a critical condition, requiring high doses of pressors. Three of four sets of blood cultures obtained on admission turned positive in anaerobic bottles. A gram stain of the blood cultures showed Gram-positive rods with spores (Figure 1), which were identified as* Clostridium paraputrificum* by the available automated identification system (VITEK 2) in our laboratory. Tissue cultures of the necrotic ascending colon obtained during the extensive colectomy also yielded* C. paraputrificum*. Metronidazole was added to the initial antibiotic regimen and the patient demonstrated gradual clinical improvement. He was weaned off mechanical ventilation and was transferred to a rehabilitation facility on day 15 after admission.

## 3. Discussion

Anaerobes are a major component of the human microflora residing on mucous membranes. Although anaerobes have been increasingly recognized as important pathogens that can cause a wide range of diseases following the breakdown of mucosal barriers at different anatomic sites, studies have reported conflicting data regarding the incidence of anaerobic bacteremia.

According to studies conducted in 1970s, the rate of anaerobes in positive blood cultures was 26.3% in 1972 [1] and 21% during the period 1974–1978 at the Mayo Clinic [2]. After that, however, the rate of anaerobic bacteremia fell to 15% during 1979–1983 and further down to 10% during 1984–1988 at the same institution [2]. In 1993, Peraino et al. investigated 7,397 blood cultures. 771 of the 7,397 blood cultures yielded bacteria or fungi; 569 (7.7%) were true positive cultures, 35 (6.2%) of which yielded 48 anaerobic isolates [3]. Overall, the rate of anaerobic bacteremia appeared decreased from the 1970s through the early 1990s.

Since 1993, however, the incidence of anaerobic bacteremia has increased in a large study conducted at the Mayo Clinic. The mean annual incidence of anaerobic bacteremia was 53 cases during 1993–1996, 75 cases during 1997–2000, and 91 cases during 2001–2004, accounted for 74% increase in the total number of cases of anaerobic bacteremia per 100,000 patient-days [4]. In the same study,* Bacteroides* species, particularly* B. fragilis*, and* Clostridium* species were the most frequent isolates.

*Clostridium* spp. are Gram-positive anaerobic rods that are capable of producing endospores. Conventional classification methods for* Clostridium* spp. rely on multiple microbiological and biochemical characteristics, including gram stain morphology and carbohydrate fermentation profiles. However, the development of gene sequencing techniques made it possible to identify the genus* Clostridium* to the species level. Woo et al. conducted a study over the period 1998–2001 where all cases of clostridium bacteremia were prospectively studied and non-*perfringens* isolates were identified to the species level by 16S rRNA gene sequencing. 17 blood cultures were due to 11 non-*Clostridium perfringens* species, of which four blood cultures yielded* C. paraputrificum* [5].

*Clostridium* spp. can cause a wide spectrum of severe infections in humans, including myonecrosis, intraabdominal infections, and bacteremia. In a retrospective population-based surveillance study conducted in Canada during the period 2000–2006, 138 cases of bacteremia due to* Clostridium* spp. were found.* C. perfringens* was the most commonly identified species with 58 (42%) isolates followed by* C. septicum* (19 isolates; 14%) and* C. ramosum* (13 isolates; 9%). Only 2 cases of bacteremia were caused by* C. paraputrificum*. Significant risk factors associated with clostridial bacteremia in this study included hemodialysis, malignancy, and inflammatory bowel disease [6]. Another study investigating risk factors for mortality in patients with anaerobic bacteremia demonstrated that the presence of liver disease and patient age were the only significant risk factors in a multivariate analysis [7].

As far as we know, case reports of bacteremia due to* C. paraputrificum* are sporadic in the literature. The underlying conditions associated with bacteremia due to* C. paraputrificum* include noncyclic neutropenia [8], alcohol abuse [9], diabetes mellitus [10], sickle cell anemia [11], malignancy [12, 13], and AIDS [14]. To our knowledge, our report is the second case of* C. paraputrificum* bacteremia reported in a patient with AIDS. Importantly, isolation of certain* Clostridium* spp., such as* C. septicum*, may reflect the presence of an underlying colonic malignancy. Horie et al. reported a deleterious effect of* C. paraputrificum* on undesirable bile acid transformation, which may be associated with an increased risk of colon cancer [15]. Nevertheless, further studies are needed to determine the association between* C. paraputrificum* and colonic malignancy given the paucity of case reports and studies on* C. paraputrificum*-associated bacteremia.

## 4. Conclusions

*Clostridium* spp. are one of the most commonly identified isolates of anaerobic bacteremia. Although* C. paraputrificum* is an infrequent isolate from blood cultures, a wide range of underlying medical comorbidities have been described in the literature. We reported a rare case of bacteremia with* C. paraputrificum* in a patient with AIDS who developed colonic necrosis. Further studies are needed to elucidate the disease spectrum, pathogenesis, and risk factors for* C. paraputrificum*-related invasive infections, including bacteremia.

## References

[1] Wilson W. R., Martin W. J., Wilkowske C. J., Washington J. A. (1972). Anaerobic bacteremia. *Mayo Clinic Proceedings*.

[2] Dorsher C. W., Rosenblatt J. E., Wilson W. R., Ilstrup D. M. (1991). Anaerobic bacteremia: decreasing rate over a 15-year period. *Reviews of Infectious Diseases*.

[3] Peraino V. A., Cross S. A., Goldstein E. J. C. (1993). Incidence and clinical significance of anaerobic bacteremia in a community hospital. *Clinical Infectious Diseases*.

[4] Lassmamn B., Gustafson D. R., Wood C. M., Rosenblatt J. E. (2007). Reemergence of anaerobic bacteremia. *Clinical Infectious Diseases*.

[5] Woo P. C. Y., Lau S. K. P., Chan K.-M., Fung A. M. Y., Tang B. S. F., Yuen K.-Y. (2005). Clostridium bacteraemia characterised by 16S ribosomal RNA gene sequencing. *Journal of Clinical Pathology*.

[6] Leal J., Gregson D. B., Ross T., Church D. L., Laupland K. B. (2008). Epidemiology of *Clostridium* species bacteremia in Calgary, Canada, 2000–2006. *Journal of Infection*.

[7] Wilson J. R., Limaye A. P. (2004). Risk factors for mortality in patients with anaerobic bacteremia. *European Journal of Clinical Microbiology and Infectious Diseases*.

[8] Shandera W. X., Humphrey R. L., Stratton L. B. (1988). Necrotizing enterocolitis associated with *Clostridium paraputrificum* septicemia. *Southern Medical Journal*.

[9] Nachamkin I., DeBlois G. E. G., Dalton H. P. (1982). Clostridium paraputrificum bacteremia associated with aspiration pneumonia. *Southern Medical Journal*.

[10] Rathbun H. K. (1968). Clostridial bacteremia without hemolysis.. *Archives of Internal Medicine*.

[11] Brook I., Gluck R. S. (1980). Clostridium paraputrificum sepsis in sickle cell anemia. *Southern Medical Journal*.

[12] Bodey G. P., Rodriguez S., Fainstein V., Elting L. S. (1991). Clostridial bacteremia in cancer patients: a 12-year experience. *Cancer*.

[13] Denamur E., Tumerelle E., Lallement P. Y., Darchis J. P., Veyssier P. (1984). *Clostridium paraputrificum* fulminant septicemia and gas gangrene disclosing acute promyelocytic leukemia. *LARC Medical*.

[14] Nerad J. L., Pulvirenti J. J. (1996). *Clostridium paraputrificum* bacteremia in a patient with AIDS and Duodenal Kaposi's sarcoma. *Clinical Infectious Diseases*.

[15] Horie H., Kanazawa K., Okada M., Narushima S., Itoh K., Terada A. (1999). Effects of intestinal bacteria on the development of colonic neoplasm: an experimental study. *European Journal of Cancer Prevention*.

